# Supplementary material for: Toxicopathological studies on the effects of T-2 mycotoxin and their interaction in juvenile goats
Source: PLoS One. 2020 Mar 26;15(3):e0229463. doi: 10.1371/journal.pone.0229463 (PMC7098593; doi:10.1371/journal.pone.0229463)
Supplement: Attachment — Submitted filename: reviewer comments.docx [file pone.0229463.s001.docx]

The authors thank the evaluation team for providing a chance to modify the manuscript to meet the journal standards and improve the quality of our article.

**As per the instructions by the editor,** the complete manuscript has been carefully proof-read to minimize the typological, grammatical and bibliographical errors. The segments that appeared copied have also been re-written to minimize plagiarism. The comments given by editor have also been carefully addressed. Below are the responses to those comments:

i) **whether any animals died without euthanasia and, if so, how many**

The authors would like to inform that none of the animals died during the course of the study before euthanization.

ii) **the specific criteria used to determine whether animals should be euthanized prior to the experimental endpoint, and whether any animals met these criteria (if so, how many)**

None of the animals were sacrificed before reaching the end point of the study for any reasons.

iii) **the frequency of animal health and welfare monitoring**

The goats were housed in experimental house with sufficient day light exposure. Prior to trial, goats were dewormed and monitored for their internal parasites. Prior approval of animal ethics committee and CPCSEA, New Delhi was obtained for this experiment. ICAR-CIRG, Mathura is conducting research on goats and its National institute. all the experimental animals were regularly and daily monitored for their health and welfare. The goats were provided with sufficient quantities of feed and fresh drinking water daily. Cleaning of pens and open paddock area was provided for exercise. After one week of acclimatization, the toxin feeding trail was undertaken

iv) **the method of euthanasia**

the goats were euthanized by intravenous administration of 20% sodium pentobarbital into the jugular vein with minimal pain and suffering. The same has been provided in the manuscript also.

v) **Please revise the manuscript to rephrase the duplicated text, cite your sources, and provide details as to how the current manuscript advances on previous work.**

We wish to bring to your kind attention that the entire manuscript has been re-written at all sections in order to minimize plagiarism.

Previous reports have targeted the effects of the T-2 toxin on mice, rats, rabbits, cattle and pigs. However, this is the first report of the effects of this toxin on goats. The study has been carried out in the institute exclusively researching on goats.

**All authors thank the editor and the reviewers for their valuable suggestions for improving the total quality of the manuscript.**

Thank you

Regards
